# ‘Open Source’ Opportunities for Enhanced Collaboration in Psychotherapy Science

**DOI:** 10.32872/cpe.6569

**Published:** 2021-06-18

**Authors:** Conal Twomey, Richard Cody, John A. Johnson, Gary O’Reilly

**Affiliations:** 1 Health Service Executive, Dublin, Ireland; 2 Púca Technologies Limited, Dublin, Ireland; 3 Department of Psychology, Pennsylvania State University, State College, PA, USA; 4 School of Psychology, University College Dublin, Dublin, Ireland

According to Marvin Goldfried, psychotherapy remains an infant science characterised by a lack of consensus surrounding core and basic principles, research-practice disparity, and excessive theory-reinvention by competing schools of therapy (Goldfried, 2020). Goldfried’s concerns together point to suboptimal collaboration within the psychotherapy research community. In our view, collaboration could be improved through the wider application of ‘open source’ software development principles (e.g., open access, free distribution, and unconstrained modification) to psychotherapy science.

The origins of open source illustrate its promotion of collaboration. Initially, software products were invariably perfected ‘behind-closed-doors’ before being released as copyrighted products. In the mid-1990s, however, the Internet enabled a new way of working: members of online developer communities started to freely share modifiable software source code with each other, leading to the creation of open and free networks of online collaboration (Raymond, 1999), and subsequently to the production of several high-quality software and Internet products (e.g., Linux and Wikipedia) and mainstream adoption across industries.

Like open source, science is—at its best—an open, collaborative endeavor (Johnson, 2014). It is therefore unsurprising that open source has increasingly infiltrated science in recent years, most notably in the ‘open science’ movement, which promotes methodological transparency and open access to data and research outputs (Vicente-Saez & Martinez-Fuentes, 2018); but also in the production of laboratory equipment (Pearce, 2014), off-patent medications (Woelfle et al., 2011), and psychometric questionnaires (Dworak et al., 2021; Goldberg et al., 2006). Regarding psychotherapy, journals routinely promote open-science practices, data from psychotherapy studies are often shared (e.g., in patient level meta-analyses), many outcome measures are freely available online, and there are an increasing number of open research networks.

Regrettably given their potential to enhance the open collaboration inherent in good science, there exist few applications of open source principles to the development of psychotherapy interventions. Most intervention manuals are not freely available online, limiting access and creating a financial barrier to the exploration of manuals from different schools of therapy. Moreover, for the vast majority of psychotherapies, copyright control and vested interests discourage (a) the collaborative modification and distribution of new versions of intervention manuals, and (b) the collaborative combination of components from different schools of therapy into transtheoretical interventions, or ‘process-based therapies’ (Hofmann & Hayes, 2019).

Regarding (a), such collaboration could be enabled if freely *modifiable* versions of intervention manuals were periodically released on open source platforms such as the Open Science Framework (https://osf.io). This would signpost progress and later facilitate the empirical comparison of different versions, in turn facilitating ‘component analyses’ that tap into basic principles. On a cautionary note, there is potential for the misuse of open source intervention manuals by unqualified persons and this should be closely monitored (Goldberg et al., 2006). Regarding (b), the vested interest of a school of therapy is to keep the learner within their school, so that the learner can eventually graduate as a proponent of the school’s teachings; however, the wider community interest is to build unifying theories that transcend the teachings of particular schools (Goldfried, 2020). Transtheoretical open source interventions provide a means for this theory unification.
